# Platelet-derived growth factor and transforming growth factor beta synergistically potentiate inflammatory mediator synthesis by fibroblast-like synoviocytes

**DOI:** 10.1186/ar2981

**Published:** 2010-04-09

**Authors:** Sanna Rosengren, Maripat Corr, David L Boyle

**Affiliations:** 1 Division of Rheumatology, Allergy and Immunology, University of California at San Diego School of Medicine, 9500 Gilman Drive, La Jolla, CA 92093-0656, USA

## Abstract

**Introduction:**

The objective of this study was to model the effects of transforming growth factor beta (TGF-β) and platelet-derived growth factor (PDGF), both present in rheumatoid arthritis (RA) synovia, on the behavior of fibroblast-like synoviocytes (FLS) in response to pro-inflammatory cytokine (interleukin (IL)1β, tumor necrosis factor-alpha (TNFα)) challenge.

**Methods:**

Gene and protein expression by fibroblast-like synoviocytes *in vitro *was studied by quantitative Polymerase Chain Reaction (qPCR), ELISA and multiplex bead cytokine assays. Intracellular signaling pathway activation was determined by Western blot for phospho-kinases and the use of specific inhibitors.

**Results:**

In combination, TGF-β and PDGF (2GF) synergistically augmented TNFα- or IL1β-induced matrix metalloproteinase 3 (MMP3), IL6, IL8, and macrophage inflammatory protein 1 alpha (MIP1α) secretion by FLS. Other FLS-derived mediators remained unaffected. Individually, neither growth factor significantly potentiated TNFα or IL1β-induced MMP3 secretion, and only slightly enhanced IL6. The effect of 2GF on TNFα-induced gene expression was transcriptionally mediated; blocked by imatinib mesylate; and occurred even if 2GF was added as much as four hours prior to TNFα. In addition, a 15-minute pulse of 2GF four hours prior to TNFα stimulation yielded a synergistic response. The extracellular-signal-regulated kinase (ERK) and phosphoinositide 3-kinase (PI3K) signaling pathways were induced for at least four hours by 2GF, as demonstrated by persistently upregulated levels of phospho-Akt and phospho-ERK. However, pharmacologic inhibitor studies demonstrated that the potentiating action of 2GF was dependent on PI3 kinase only, and not on ERK.

**Conclusions:**

The combination of PDGF and TGF-β dramatically potentiates FLS response to cytokines in a receptor-mediated and PI3 kinase-dependent fashion. These data suggest that 2GF contribute to synovitis by directing synovial fibroblasts toward a more aggressive phenotype in response to TNFα. Therefore, inhibition of growth factor signaling may constitute a complementary therapeutic approach to cytokine-targeted treatments for RA.

## Introduction

Expression of the regulatory peptides, platelet-derived growth factor (PDGF) and transforming growth factor beta (TGF-β) are increased in synovial tissue and fluid of rheumatoid arthritis (RA) patients [1-4]. PDGF has been implicated in RA pathogenesis, mainly through its function as a growth factor for fibroblast-like synoviocytes (FLS) [3,5]. In contrast, the actions of TGF-β are more complex. TGF-β plays a crucial role in maintaining immunological tolerance through the inhibition of lymphocytes and macrophages [6]. On the other hand, it recruits and activates naive monocytes [6], stimulates proliferation [7] and induces aggrecanase synthesis [8] by FLS. Systemic administration of TGF-β protects against development of collagen arthritis in mice [9], whereas direct injection of TGF-β into rat joints leads to pronounced synovitis [10].

In addition to these growth factors, chronically inflamed RA synovia contain a multitude of inflammatory mediators that may act in concert with each other. In this context, aggravating as well as mitigating effects of growth factors and cytokines on FLS have been demonstrated. For example, PDGF was reported to enhance IL1β-induced prostaglandin E2 production, while inhibiting collagenase synthesis [11]. Also, PDGF was shown to induce synthesis of IL8 and MIP1α, along with IL1β, by FLS [12], and also to synergize with TNFα to stimulate IL1β secretion, although these results are somewhat confusing since FLS are not typically considered a significant source of IL1β. On the other hand, TGF-β was earlier shown to inhibit TNFα-induced RANTES synthesis by FLS [13]. A systematic study of the nature of the interaction among these mediators was not undertaken to date. Hence, the interplay between PDGF, TGF-β, and cytokines such as TNFα and IL1β on the activation of FLS remains unclear, albeit of potential significance considering the abundance of these proteins in the RA synovial environment.

Consequently, we set out to systematically determine the effect of PDGF and TGF-β, alone and in combination, on inflammatory biomarker expression and secretion by FLS. We describe significant potentiation by PDGF and TGF-β of the production of certain cytokines, chemokines, and matrix metalloproteinases (MMP) by FLS. This synergy was mediated by tyrosine-kinase receptor activation and dependent on PI3K, both of which are receiving attention as possible novel approaches to RA drug therapy.

## Materials and methods

### Reagents

Cytokines and TGF-β were obtained from R&D Laboratories (Minneapolis, MN, USA). Imatinib mesylate (LC Laboratories, Woburn, MA, USA) was dissolved in water. All other reagents, including PDGF-BB, were from Sigma (St. Louis, MO, USA) unless otherwise noted. Stock solutions in DMSO (1000×) of PD98059 and LY294002 were kept at -80°C.

### Fibroblast-like synoviocytes (FLS)

FLS were cultured from the synovial tissues of RA patients undergoing arthroplastic surgery, as previously described [14], after obtaining informed consent under approval from the University of California, San Diego Institutional Review Board, and maintained in Dulbecco's Modified Eagle Medium (DMEM) supplemented with antibiotics, glutamine, and 10% fetal bovine serum. Passages 4 through 8 were used in experiments. Cells were subjected to a two- to three-day reduced serum condition (0.1% fetal bovine serum) prior to stimulation to minimize baseline activity.

### Secreted protein assays

FLS supernatants at 24 hours following stimulation were assayed by ELISA for IL6 (eBioscience, San Diego, CA, USA), MMP1, and MMP3 (GE Healthcare Life Sciences, Piscataway, NJ, USA). Standard curves were constructed by regression line fitting on log(absorbance) vs log(concentration). Levels of cytokines and chemokines in supernatants were determined by Luminex multiplex analysis (BioRad Bio-Plex assays, Hercules, CA, USA) from four-parameter standard curve fits.

### Gene expression assays

Messenger RNA for IL6, MIP1α, and MMP3 were quantified by real-time TaqMan quantitative Polymerase Chain Reaction (qPCR), using FLS cDNA, with GAPDH used as a housekeeper (all reagents from Applied Biosystems, Foster City, CA, USA). Resulting threshold cycle (Ct) data were normalized to standard curves constructed from cDNA from IL1β-stimulated FLS [15], yielding cell equivalents. The ratio between the specific cytokine and GAPDH cell equivalents (relative expression units, REU) is reported.

### Western blot

FLS extracts were prepared in RIPA buffer with Complete Protease Inhibitors (Roche Applied Science, Indianapolis, IN, USA), denatured in sample buffer and 0.1 M dithiotreitol, and fractioned on Invitrogen (Carlsbad, CA, USA) NuPage 4 to 12% precast gels. Following blotting to polyvinylidene fluoride (PVDF) membranes and blocking with 5% dry milk, blots were probed with antibodies against phospho- or total p38, JNK, Erk, or Akt, as well as with secondary anti-rabbit-IgG-HRP (all Cell Signaling Technologies, Danvers, MA, USA). GAPDH was used as a gel loading control (antibody from Santa Cruz Biotechnology, Santa Cruz, CA, USA). Membranes were developed with Immun-Star WesternC ECL substrate (BioRad, Hercules, CA, USA) and imaged on a VersaDoc imaging system (BioRad), using QuantityOne software (Hercules, CA, USA) for image capture and densitometry.

### Statistical analysis

Data are reported as mean and standard error of the mean (SEM). Protein secretion and gene expression data in single time-point experiments were analyzed by one-way ANOVA followed by Tukey-Kramer's post-hoc test comparing all groups, or by Dunnett's post-hoc test comparing control to all others, as appropriate. Time course data were analyzed by two-way ANOVA followed by contrast testing. Student's t-test was used to examine synergistic effects of growth factors and cytokines. Real-time qPCR data were log-transformed prior to analysis.

## Results

### Effect of PDGF-BB and TGF-β on FLS secretion of inflammatory mediators

**Figure 1:**
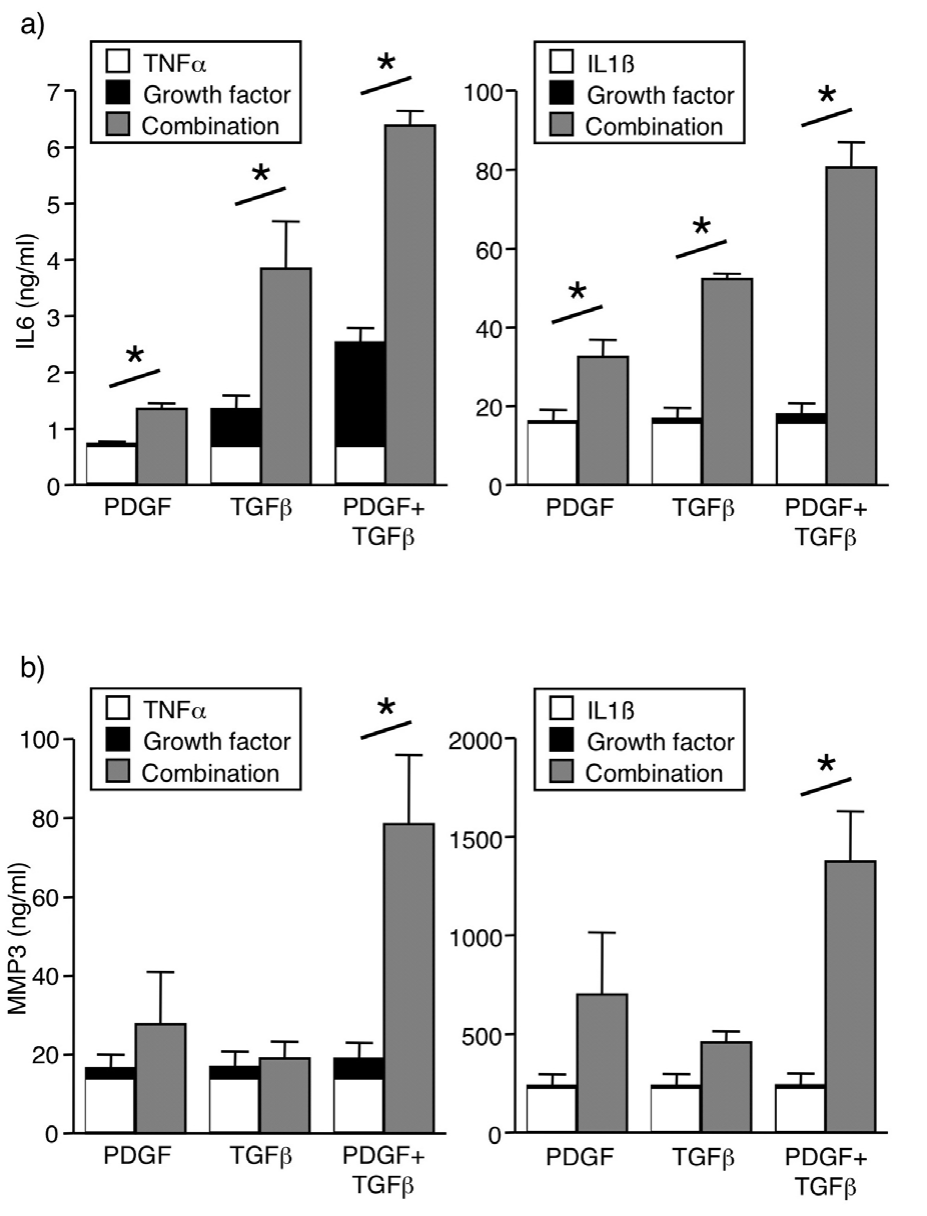
**Potentiation by PDGF alone, TGF-β alone, or their combination (2GF), of (a) IL6 and (b) MMP3 secretion from FLS**. FLS were cultured for 24 hours with TNFα (10 ng/ml) or IL1β (2 ng/ml), and/or growth factors (10 ng/ml), and supernatants analyzed by ELISA. Mean & SEM, n = 3 RA FLS lines. Asterisk indicates *P *< 0.05 between the combination and the added values for TNF alone and growth factor alone by Students' t-test.

**Figure 2:**
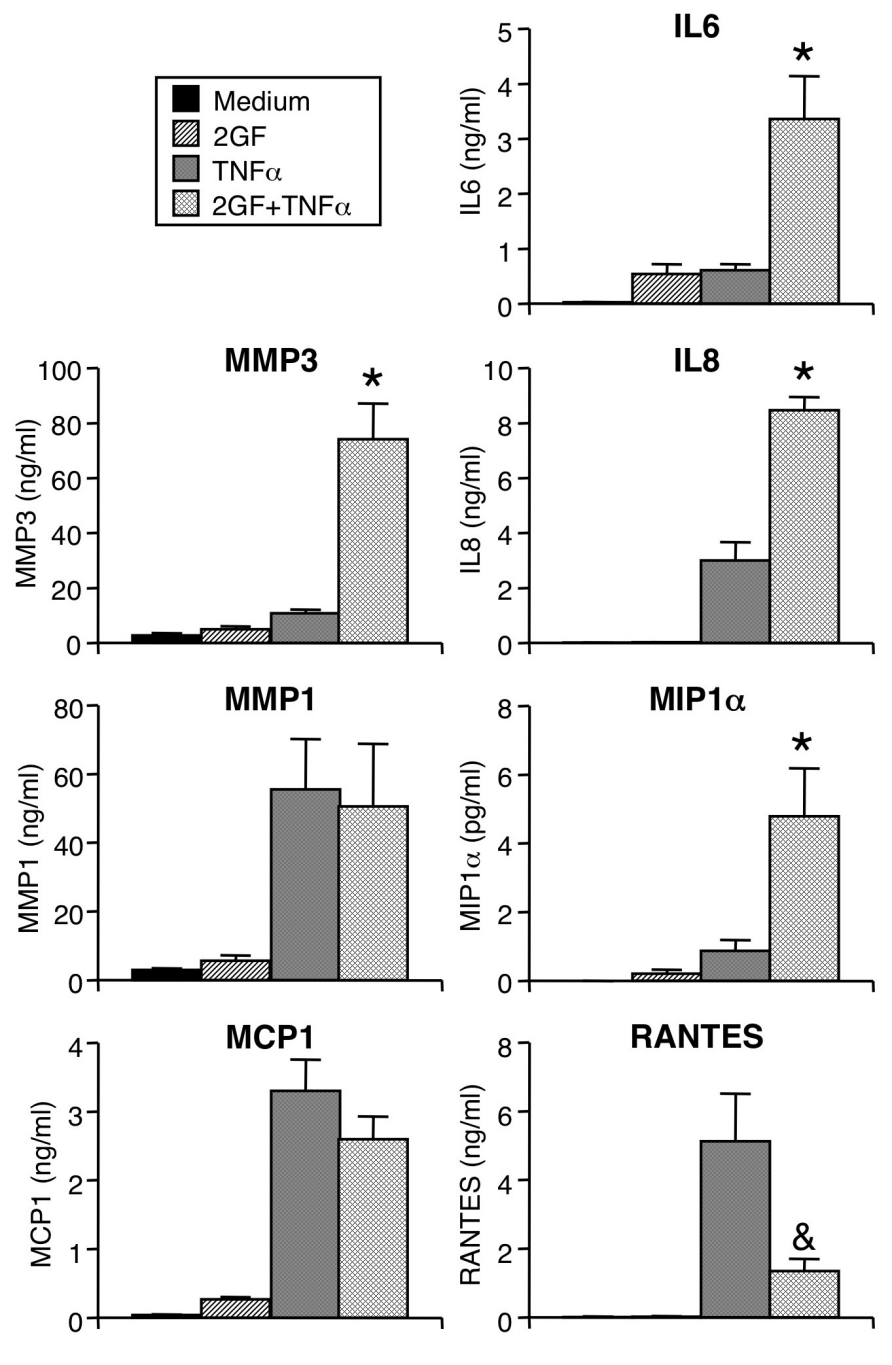
**Augmentation by 2GF of FLS secretion of particular cytokines, chemokines and MMPs induced by TNFα**. FLS were cultured for 24 hours with TNFα and growth factors as in Figure 1, and supernatants analyzed by ELISA (MMPs) or Luminex multiplex bead assay (all others). Mean & SEM, n = 3 to 6 RA FLS lines. Asterisk indicates *P *< 0.05 to TNFα alone and 2GF alone, and ampersand indicates *P *< 0.05 to TNFα alone, by ANOVA/Tukey-Kramer's.

Since PDGF and TGF-β are abundant in the rheumatoid synovium, their effect on cytokine-induced inflammatory mediator secretion by FLS was examined. TGF-β induced only a small amount of IL6 (Figure 1a), and no effect on IL6 (Figure 1a) or MMP3 (Figure 1b) was observed by PDGF-BB alone. PDGF and TGF-β in combination (2GF) induced low-level secretion of IL6, but not MMPs or chemokines (Figures 1 and 2). The amount of IL6 secreted after 2GF stimulation was comparable to that observed with TNFα as the stimulant (Figure 2).

Surprisingly, the two growth factors in combination potently augmented secretion of IL6 (Figure 1a) and MMP3 (Figure 1b) in response to TNFα or IL1β. The effect of 2GF was truly synergistic, in that the secretion observed by 2GF and TNFα or IL1β in combination was significantly higher than that obtained when adding the values for 2GF alone and cytokine alone (Figure 1). When PDGF-BB and TGF-β were examined individually, neither augmented TNF- or IL1β-induced MMP3 secretion, and the effect on TNF- or IL1β-induced IL6 secretion was smaller than that of the growth factor combination (Figure 1). The potentiating effect of 2GF was not simply due to a non-specific effect of cell activation, since the secretion of some but not all mediators was affected. TNFα-induced secretion of MMP1 and MCP1 was unaltered by addition of 2GF, and RANTES secretion was inhibited, at the same time that IL8 and MIP1α secretion was potentiated (Figure 2) along with that of IL6 and MMP3.

**Figure 3:**
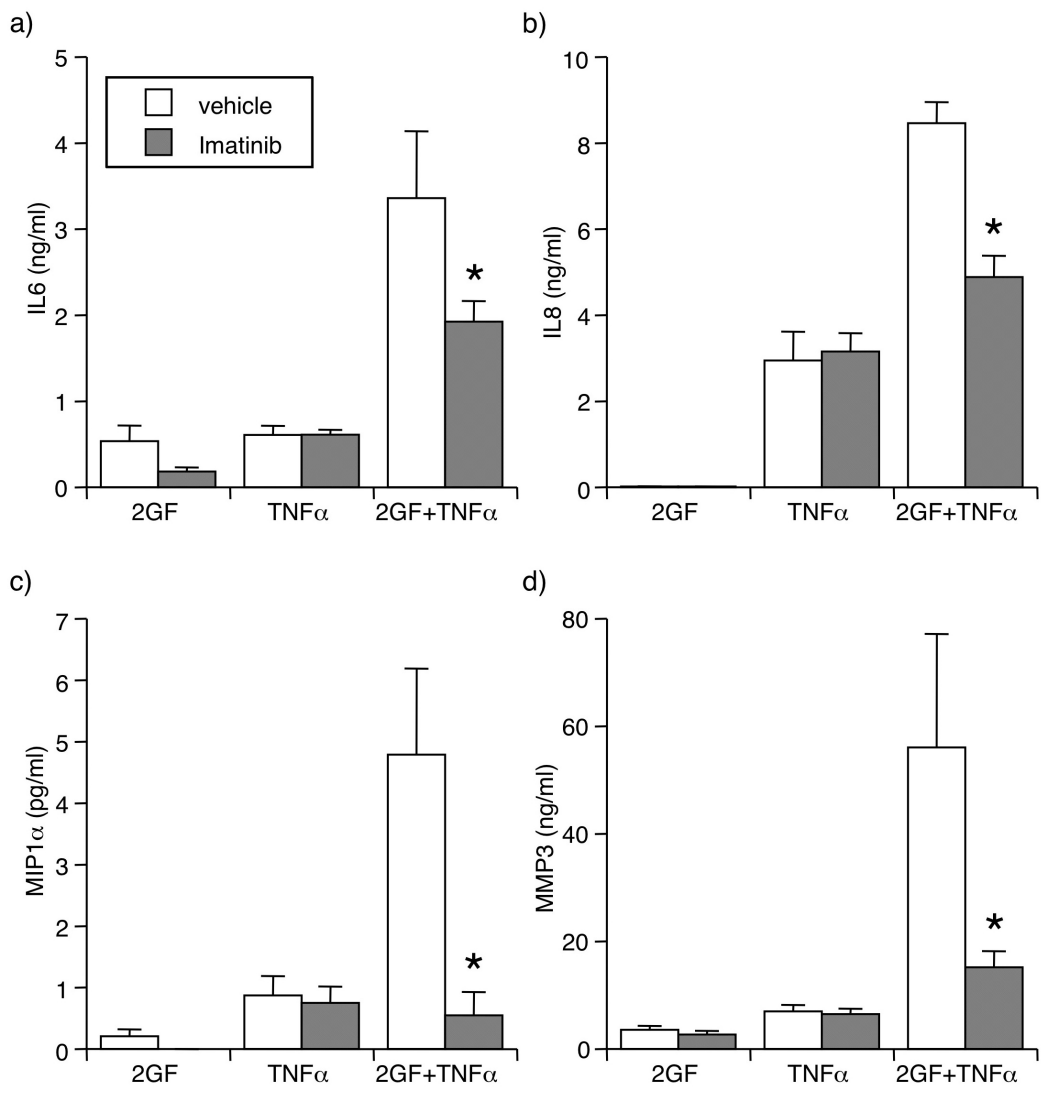
**Reversal by imatinib (1 μM) of 2GF potentiation of TNFα-induced (a) IL6, (b) IL8, (c) MIP1α, and (d) MMP3 secretion**. For culture conditions and definitions, see legends for Figure 1 and 2. Supernatants were analyzed by ELISA or Luminex multiplex bead assay. Mean & SEM, n = 3 RA FLS lines. Asterisk indicates *P *< 0.05 to vehicle by Students' t-test.

The effect of 2GF was mediated through activation of growth factor receptors, since the receptor tyrosine kinase inhibitor, imatinib mesylate significantly reversed the potentiating effect of 2GF on TNFα-induced secretion of IL6, IL8, MIP1α, and MMP3 (Figure 3). Importantly, imatinib did not alter secretion of these mediators in response to TNFα alone.

### Effect of PDGF-BB and TGF-β on the time course of FLS mRNA expression

**Figure 4:**
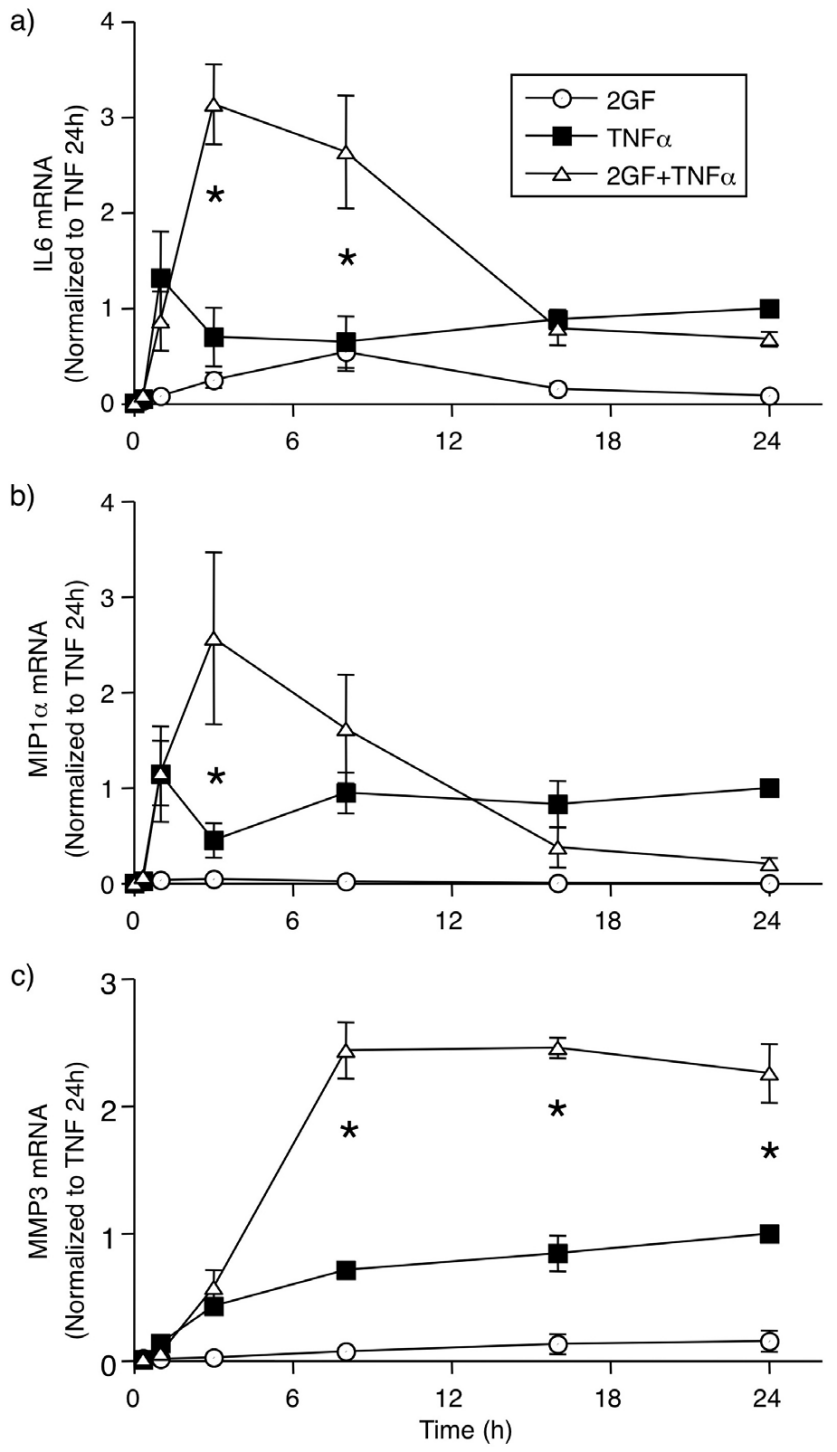
**Time course of 2GF-induced potentiation of (a) IL6, (b) MIP1α and (c) MMP3 RNA induced by TNFα**. FLS were cultured for indicated times with TNFα and growth factors, and mRNA levels quantified by real-time qPCR using GAPDH as housekeeper. Data are normalized to levels with TNFα alone at 24 h. Mean & SEM, n = 3 RA FLS lines. Asterisk indicates *P *< 0.05 to TNF alone and 2GF alone by two-way ANOVA and contrast testing on log-transformed data.

In order to determine whether the effect of 2GF on FLS protein secretion was observed at the mRNA expression level, a time course experiment was conducted and the expression of IL6, MIP1α, and MMP3 mRNA in FLS was studied. TNFα caused a rapid rise in IL6 (Figure 4a) and MIP1α (Figure 4b) mRNA expression, reaching a plateau at one hour and maintaining significant expression until the end of the experiment at 24 h. 2GF alone induced a small amount of IL6 mRNA at three and eight hours, but no MIP1α. When 2GF and TNFα was added in combination, significantly elevated IL6 levels were observed at three and eight hours (Figure 4a). For MIP1α (Figure 4b), potentiation by 2GF of TNFα-induced chemokine was only observed at three hours. Similar results were obtained for IL8 expression (data not shown). In the case of MMP3, TNFα alone induced a slow steady increase of mRNA levels evident from three hours and lasting until the end of the experiment at 24 h. The addition of 2GF in combination with TNFα led to significantly elevated MMP3 levels at 8, 16 and 24 h (Figure 4c). Thus, the synergistic effect of 2GF on TNFα-induced inflammatory mediator production by FLS is evident at the transcriptional level.

### Effect of temporal separation of the addition of growth factors and TNFα to FLS

**Figure 5:**
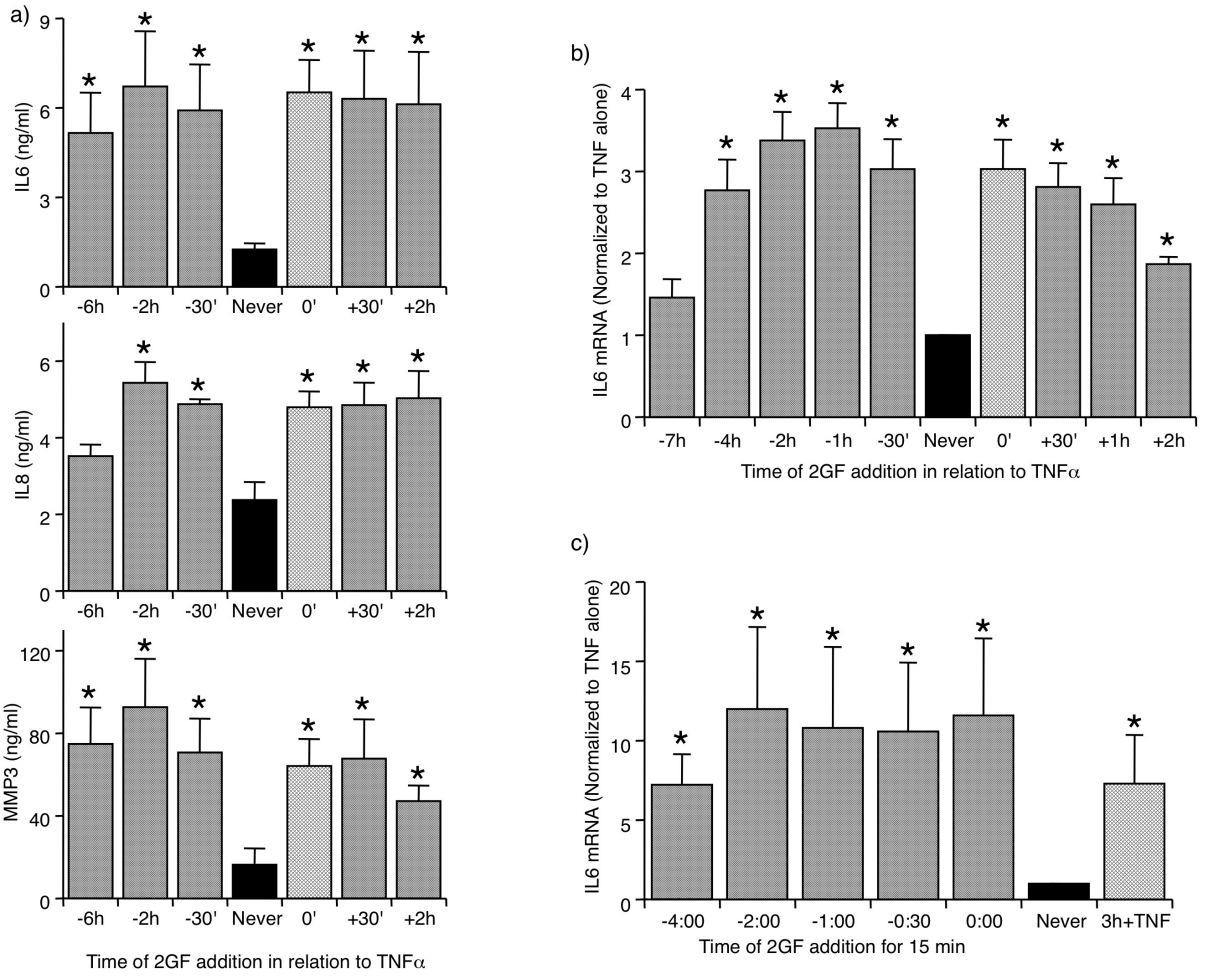
**Synergy tolerates temporal separation of 2GF and TNFα stimulation of FLS**. 2GF was added at the indicated timepoint, and left on for the remainder of the experiment (**a-b**) or removed after 15 minutes **(c) **(shaded bars). TNFα was added at time zero, and supernatants harvested at +24 h (a) or RNA isolated at +3 h (b-c). As controls, results with TNFα alone (black bar, *Never*) and TNF+2GF added simultaneously and left on (cross-hatched bar) are shown. Data are normalized to levels with TNFα alone. Mean & SEM, n = 3 RA FLS lines. Asterisk indicates *P *< 0.05 to TNFα alone by ANOVA/Dunnett's on raw (a) or log-transformed (b-c) data.

Next, the addition of 2GF and TNFα was separated in time to determine whether the potentiating effect of 2GF would be maintained. PDGF and TGF-β were added at various time points in relation to TNFα, which was in turn allowed to stimulate the FLS for 24 h before supernatants were analyzed for secreted proteins. Under these conditions, 2GF was able to potentiate TNFα-induced IL6, IL8 and MMP3 secretion when added at any time between -2 h and +2 h in relation to a TNFα addition (Figure 5a). The extent of the potentiating effect was similar to that observed when 2GF and TNFα were added simultaneously (crosshatched bars). For IL6 and MMP3 secretion, potentiation by 2GF was also observed when added as much as six hours prior to TNFα (Figure 5a).

In similar experiments studying the gene mRNA expression at three hours following TNFα addition, 2GF synergistically potentiated TNFα-induced IL6 expression when added between -4 h and +2 h in relation to TNFα addition (Figure 5b). In separate experiments, FLS could be exposed to 2GF for as little as 15 minutes, even when added as early as four hours before TNFα, and significantly elevated IL6 expression could still be noted (Figure 5c). This suggests that the synergistic effect does not require continuous exposure to the 2GF, and that it involves signaling pathways that are maintained over the course of several hours.

### Sustained activation of Erk and Akt in FLS by growth factors

**Figure 6:**
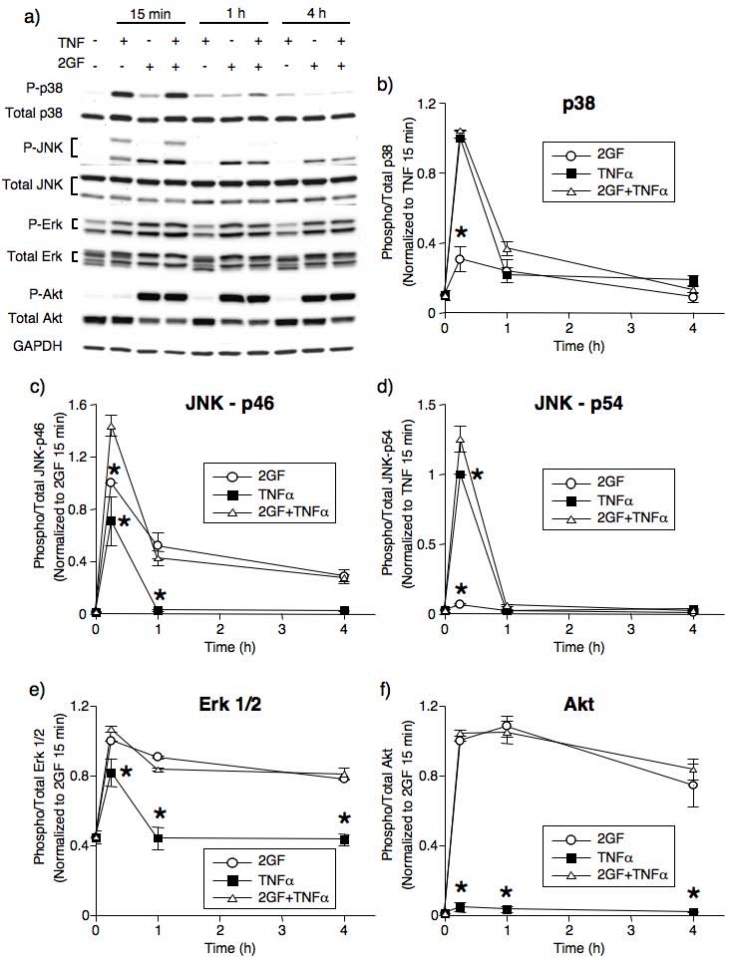
**Time course of phospho-protein induction by TNFα, 2GF, or a combination in FLS**. **(a)**. Representative Western blot scans. GAPDH was used as loading control. **(b)-(f)**. Ratios between phospho- and total MAPK and Akt as determined by densitometry. The two ERK isoforms were analyzed together (e). Data are normalized to 2GF or TNFα alone at 15 minutes, as indicated. Mean & SEM, n = 3 RA FLS lines. Asterisk indicates *P *< 0.05 to 2GF+TNFα by two-way ANOVA and contrast testing.

For the purpose of elucidating the relevant signaling pathways causing the synergistic effect, FLS were treated with TNFα, 2GF, or a combination for 15 minutes to four hours, and cell extracts analyzed by Western blot (Figure 6a). TNFα induced a short-lived peak of phosphorylation of p38, JNK isoforms, and ERK isoforms (Figure 6b-e) but had a marginal effect on Akt phosphorylation (Figure 6f). In contrast, 2GF induced a different pattern: phosphorylation of ERK and Akt that lasted for the four hours studied (Figure 6e-f), no phosphorylation of p38 (Figure 6b) nor JNK-p54 (Figure 6d), and a short-lived upregulation of phospho-JNK-p46 (Figure 6c). In combination, 2GF and TNFα generated phospho-protein levels similar to those induced by the mediators added separately, with the sole exception of phospho-JNK which was significantly higher after 15 minutes of 2GF + TNFα than after TNF alone or 2GF alone (Figure 6c, d). At the four-hour time point, no synergistic effect of 2GF and TNFα was noted on any phospho-protein studied. These studies suggest focusing on the PI3K and MEK/ERK pathways as potentially responsible for the synergy.

### Effect of pharmacological inhibitors on 2GF potentiation of IL6 mRNA expression by FLS

**Figure 7:**
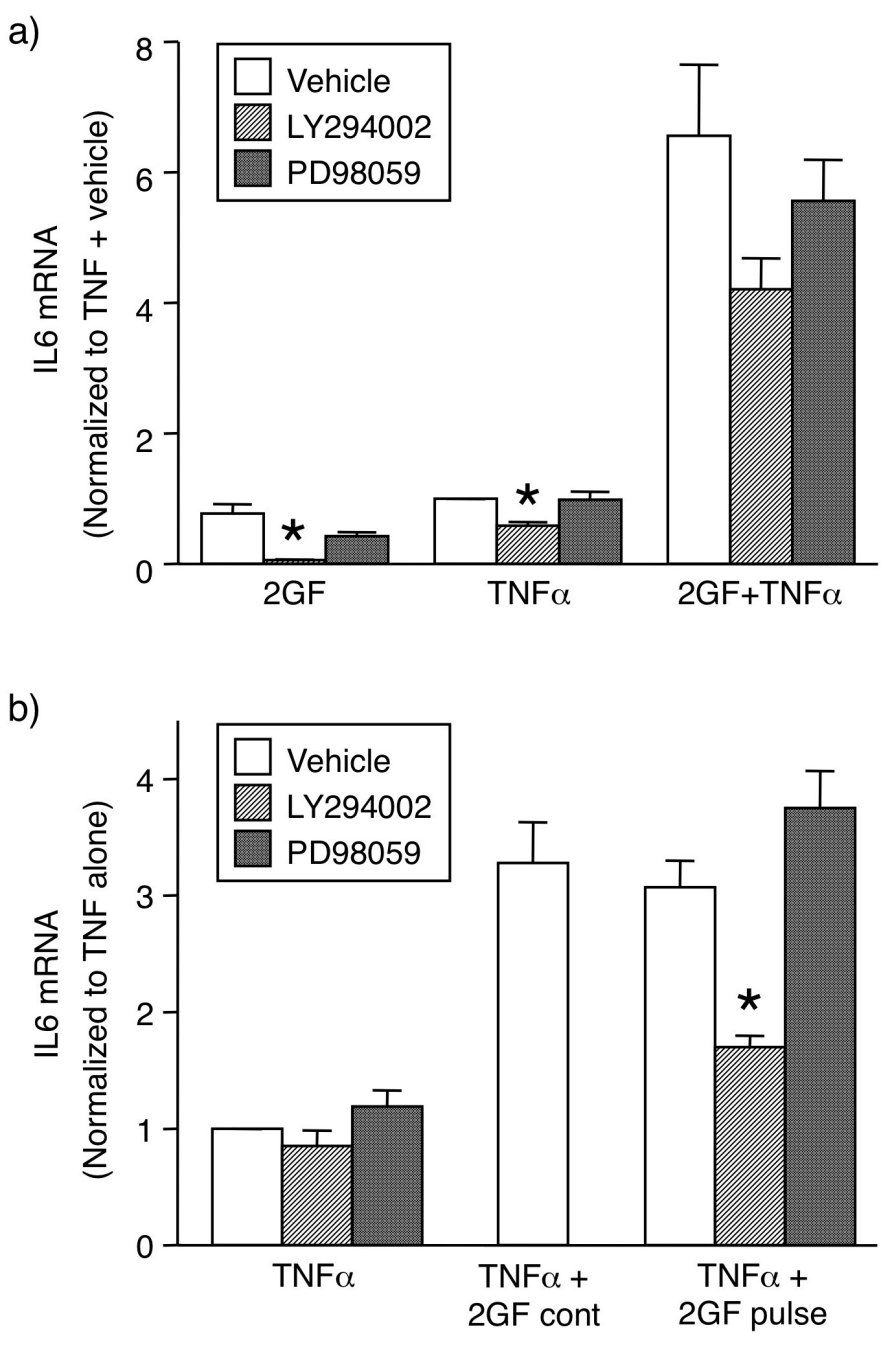
**Involvement of PI3 kinase, but not Erk, in 2GF potentiation of TNFα-induced IL6 gene expression by FLS**. **(a)**. FLS were pre-treated with PD98059 (20 μM) or LY294002 (40 μM) 30 minutes prior to 2GF and TNFα simultaneous addition. **(b)**. Inhibitors were added at -4 h 30 minutes, 2GF -4 h, and TNFα at time zero. 2GF were left on for the duration of the experiment (*2GF cont*) or left on for 15 minutes only (*2GF pulse*). RNA was isolated at three hours following TNFα stimulation. Data are normalized to levels with TNFα + vehicle. Asterisk indicates *P *< 0.05 to respective vehicle by ANOVA/Dunnett's (a) or ANOVA/Tukey-Kramer's (b) on log-transformed data.

We tested the relative contributions of the ERK and PI3K signaling cascades to the synergistic effects of growth factors on gene expression using pharmacological inhibitors of ERK kinase (MEK1; PD98059) and PI3K (PI3Kα, PI3Kβ, PI3Kγ and PI3Kδ; LY294002). When 2GF and TNFα were added simultaneously in the presence of inhibitors, PD98059 had no effect on IL6 expression induced by any stimuli (Figure 7a). In contrast, the PI3K inhibitor, LY294002 had a significant effect on the IL6 expression induced by 2GF alone or TNFα alone, but in the case of the combination the effect, although evident, did not reach statistical significance (Figure 7a).

Since the interpretation of these results were complicated by the fact that LY294002 significantly inhibited the response to TNFα alone, 2GF were added to FLS cultures for 15 minutes only, and then soluble 2GF was removed by a medium change. Four hours later, TNFα was added and allowed to stimulate the FLS for a total of three hours, similar to the experiments shown in Figure 5c. The potentiating effect induced by 2GF under these conditions was significantly reversed if the PI3K inhibitor, LY294002, was included prior to the 2GF pulse (Figure 7b). In this study, LY294002 had no effect on the IL6 expression induced by TNFα alone in these experiments (Figure 7b), thus demonstrating that the effect was specific to 2GF-induced PI3K activity. Since the ERK pathway inhibitor had no effect in this system, these results indicate that activation of the PI3K pathway is a crucial step for the 2GF potentiation of TNFα-induced gene expression in FLS.

## Discussion

The chronically inflamed rheumatoid synovium is a complex environment with various cellular subtypes, cytokines, growth factors, chemokines, proteases and mechanical phenomena interacting with each other over time. Animal models may provide valuable insights into disease processes, but are limited in their ability to demonstrate specific target mediated effects that correspond to observations in RA. In addition, the typical rat and mouse models utilized, albeit useful in many ways, do not fully recapitulate human disease [16]. Studies of synovial tissue *ex vivo *can provide a snapshot of cellular activity in RA, and the accumulation of these observations provide insight into disease pathogenesis. *In vitro *studies of isolated human synovial cells can illuminate dynamic disease-specific cellular mechanisms. However, complete recapitulation of the RA synovial complexity *in vitro *is impractical if not impossible. Typical *in vitro *studies involve stimulating or activating cells, blocking signaling pathways and observing disease-relevant gene expression or proliferative outcomes. Interestingly, such studies have demonstrated what appear to be unresolved opposing effects of various mediators known to be present in the rheumatoid synovium. In this study we attempt to incrementally close the gap between cells and tissue by evaluating the role of peptide mediators historically identified as growth factors (PDGF and TGF-β) in providing a context for the response of FLS to inflammatory cytokines.

The surprising and novel central finding of these studies is the significant and striking synergistic effect of a combination of PDGF and TGF-β on cytokine-induced FLS secretion of selected inflammatory mediators (IL6, IL8, MIP1α and MMP3), while leaving some other mediators unaltered. Both PDGF and TGF-β induce proliferation of FLS [3,5,7], and cytokine-induced growth of FLS is potentiated by PDGF [17] and TGF-β [7]. Therefore, a potential reason for the synergistic effect of growth factors and cytokines on secretion of inflammatory mediators by FLS could simply be that a higher number of FLS are present after growth factor activation. This is unlikely to provide an explanation for our findings, however, for two reasons. First, FLS are slow growing cells [14] and the relatively short incubation times employed in the current studies (3 h for mRNA, 24 h for protein secretion) make it unlikely that a significantly higher number of FLS could have been generated. Second, in the mRNA expression studies, all data were normalized to GAPDH for the purpose of controlling for cell numbers. Since the mRNA and protein results essentially mirrored each other (compare for example Figures 2 and 4, and Figures 5a and 5b), the underlying reason for the synergy of the two growth factors along with cytokines on FLS is unlikely to be simply an effect on cell number.

To our knowledge, this report is the first to establish a synergy of the combined effects of PDGF and TGF-β on cytokine-induced gene expression in FLS. The underlying signaling mechanisms are not entirely clear. However, the effect is receptor-mediated as demonstrated by the reversing action of imatinib mesylate, also known as Gleevec. This compound is a moderately selective tyrosine kinase inhibitor that targets several classes of receptor kinases including abl [18], c-kit [19], c-fms (the M-CSF receptor) [20], and PDGF receptor kinases [18,21]. In FLS, imatinib blocks PDGF-induced proliferation and phosphorylation of downstream targets of PDGF receptor stimulation [22-25]. Due to its inhibition of abl, imatinib also has a role in TGF-β induced signaling and fibrogenesis in cultured fibroblasts [26,27]. Hence, the reversal of the growth factor-induced synergy by imatinib indicates involvement of specific growth factor signaling pathways.

With respect to common signaling pathways in fibroblasts, both PDGF and TGF-β are known to activate the PI3K [28,29] and the Ras-Raf-MEK-ERK pathways [30,31]. Indeed, both Akt and ERK were phosphorylated for at least four hours by 2GF treatment of FLS, making them attractive signaling candidates. The testing of this hypothesis was complicated by the fact that the PI3K inhibitor used (LY294002) had significant effects on IL6 expression induced by TNFα alone, as earlier reported [32] and similar to earlier published results where IL17 was used to induce IL6 [33]. To circumvent this problem, we took advantage of the fact that a short pulse of 2GF, separated in time from the TNFα stimulation, was capable of potentiating TNFα-induced IL6 expression to the same extent as continuous incubation with 2GF without affecting signaling in FLS stimulated with TNFα alone. In this system, LY294002 added before 2GF and removed prior to the addition of TNFα significantly blocked the synergy, demonstrating a PI3K role. The ERK pathway, however, did not appear to play a role, at least at levels distal to MEK1. Thus, PI3K constitutes a pharmacological target of interest for synovitis mediated by this mechanism. Indeed, studies antagonizing PI3K signaling have shown promise in animal models of arthritis. Gene transfer of a negative regulator of PI3K signalling, PTEN, ameliorates collagen arthritis [34] and in murine models of arthritis, inhibitors of the gamma isoform PI3K have been shown to reduce joint destruction [35]. Notably, this particular isoform was recently demonstrated to be specifically upregulated in human RA FLS [36].

These findings, in addition to demonstrating novel synergistic effects of growth factors and cytokines on FLS, may also have clinical implications. In particular, the effect of imatinib is of interest, since this compound is already in clinical use for Philadelphia chromosome-positive hematological malignancies [37] as well as for gastrointestinal stromal tumor [38]. A few case reports exist [39,40] of imatinib mesylate as a successful treatment for refractory RA, with reductions in swollen joint counts and CRP observed. In addition, a phase II study of imatinib in RA has been completed (Clinicaltrials.gov identifier # NCT00154336), however the results have not yet been made publicly available. In animal models, imatinib limits joint inflammation in mouse collagen arthritis [23,41] and rat adjuvant arthritis [25], and reduces joint destruction in collagen arthritis in rats [42]. Additionally, in preliminary studies in our laboratory, imatinib limited the arthritis induced by K/BxN serum transfer (data not shown), a murine model in which the adaptive immune system has been bypassed. The precise mechanism of imatinib in RA is not known and could involve downregulation of the function of a number of cell types, as shown *in vitro*: T and B lymphocytes [23,43], macrophages [20,44], osteoclasts [42], and mast cells [23,45]. The studies described herein provide yet another potential explanation for the effect of imatinib in arthritis: inhibition of a two-legged response by FLS, which require both a cytokine and growth factors to become activated to its fullest potential.

## Conclusions

PDGF and TGF-β strongly and selectively potentiate cytokine-induced synthesis and secretion of certain pro-inflammatory factors by FLS, such as IL6, IL8, MIP1α, and MMP3. The synergy was transcriptionally regulated, and endured for at least several hours after withdrawal of the growth factors. These data are consistent with a model wherein PDGF and TGF-β direct the response of synovial cells toward an RA phenotype and may partially explain the aggressiveness of RA synovitis. Both imatinib mesylate and a PI3K inhibitor were found to reverse this synergy. Therefore, targeting growth factor signaling may provide an additional approach to breaking the cycle of sustained synovitis in RA with the goal of restoring synovial homeostasis.

## Abbreviations

2GF: both TGF-β and PDGF were used together; Ct: threshold cycle; FLS: fibroblast-like synoviocytes; IL: interleukin; MIP1α: macrophage inflammatory protein 1 alpha; MMP: matrix metalloproteinase; PDGF: platelet-derived growth factor; RA: rheumatoid arthritis; REU: relative expression units; TGF-β: transforming growth factor beta; TNFα: tumor necrosis factor-alpha.

## Competing interests

The authors declare that they have no competing interests.

## Authors' contributions

SR designed and performed the experiments and statistical analysis, and prepared the manuscript. MPC provided the animal model and edited the manuscript; and DLB conceived of the study, designed experiments, and edited the manuscript.

## References

[B1] Lafyatis R Thompson NL Remmers EF Flanders KC Roche NS Kim SJ Case JP Sporn MB Roberts AB Wilder RL Transforming growth factor-beta production by synovial tissues from rheumatoid patients and streptococcal cell wall arthritic rats. Studies on secretion by synovial fibroblast-like cells and immunohistologic localization J Immunol 1989 143 1142 1148 2663990

[B2] Remmers EF Sano H Lafyatis R Case JP Kumkumian GK Hla T Maciag T Wilder RL Production of platelet derived growth factor B chain (PDGF-B/c-sis) mrna and immunoreactive PDGF B-like polypeptide by rheumatoid synovium: coexpression with heparin binding acidic fibroblast growth factor-1 J Rheumatol 1991 18 7 13 1708827

[B3] Thornton SC Por SB Penny R Richter M Shelley L Breit SN Identification of the major fibroblast growth factors released spontaneously in inflammatory arthritis as platelet derived growth factor and tumour necrosis factor-alpha Clin Exp Immunol 1991 86 79 86 1914237 10.1111/j.1365-2249.1991.tb05777.x PMC1554160

[B4] Chu CQ Field M Abney E Zheng RQ Allard S Feldmann M Maini RN Transforming growth factor-beta 1 in rheumatoid synovial membrane and cartilage/pannus junction Clin Exp Immunol 1991 86 380 386 1747946 10.1111/j.1365-2249.1991.tb02941.x PMC1554206

[B5] Remmers EF Sano H Wilder RL Platelet-derived growth factors and heparin-binding (fibroblast) growth factors in the synovial tissue pathology of rheumatoid arthritis Semin Arthritis Rheum 1991 21 191 199 1724096 10.1016/0049-0172(91)90009-o

[B6] Li MO Wan YY Sanjabi S Robertson AK Flavell RA Transforming growth factor-beta regulation of immune responses Annu Rev Immunol 2006 24 99 146 16551245 10.1146/annurev.immunol.24.021605.090737

[B7] Wahl SM Allen JB Wong HL Dougherty SF Ellingsworth LR Antagonistic and agonistic effects of transforming growth factor-beta and IL-1 in rheumatoid synovium J Immunol 1990 145 2514 2519 2212650

[B8] Yamanishi Y Boyle DL Clark M Maki RA Tortorella MD Arner EC Firestein GS Expression and regulation of aggrecanase in arthritis: the role of TGF-beta J Immunol 2002 168 1405 1412 11801682 10.4049/jimmunol.168.3.1405

[B9] Thorbecke GJ Shah R Leu CH Kuruvilla AP Hardison AM Palladino MA Involvement of endogenous tumor necrosis factor alpha and transforming growth factor beta during induction of collagen type II arthritis in mice Proc Natl Acad Sci USA 1992 89 7375 7379 1502148 10.1073/pnas.89.16.7375 PMC49712

[B10] Allen JB Manthey CL Hand AR Ohura K Ellingsworth L Wahl SM Rapid onset synovial inflammation and hyperplasia induced by transforming growth factor beta J Exp Med 1990 171 231 247 2295877 10.1084/jem.171.1.231 PMC2187661

[B11] Kumkumian GK Lafyatis R Remmers EF Case JP Kim SJ Wilder RL Platelet-derived growth factor and IL-1 interactions in rheumatoid arthritis. Regulation of synoviocyte proliferation, prostaglandin production, and collagenase transcription J Immunol 1989 143 833 837 2545778

[B12] Cheon H Sun YK Yu SJ Lee YH Ji JD Song GG Lee JH Kim MK Sohn J Platelet-derived growth factor-AA increases IL-1beta and IL-8 expression and activates NF-kappab in rheumatoid fibroblast-like synoviocytes Scand J Immunol 2004 60 455 462 15541037 10.1111/j.0300-9475.2004.01505.x

[B13] Cho ML Min SY Chang SH Kim KW Heo SB Lee SH Park SH Cho CS Kim HY Transforming growth factor beta 1(TGF-beta1) down-regulates tnfalpha-induced RANTES production in rheumatoid synovial fibroblasts through NF-kappab-mediated transcriptional repression Immunol Lett 2006 105 159 166 16564576 10.1016/j.imlet.2006.02.003

[B14] Rosengren S Boyle DL Firestein GS Acquisition, culture, and phenotyping of synovial fibroblasts Methods Mol Med 2007 135 365 375 17951672 10.1007/978-1-59745-401-8_24

[B15] Boyle DL Rosengren S Bugbee W Kavanaugh A Firestein GS Quantitative biomarker analysis of synovial gene expression by real-time PCR Arthritis Res Ther 2003 5 R352 360 14680510 10.1186/ar1004 PMC333415

[B16] Hegen M Keith JC Jr Collins M Nickerson-Nutter CL Utility of animal models for identification of potential therapeutics for rheumatoid arthritis Ann Rheum Dis 2008 67 1505 1515 18055474 10.1136/ard.2007.076430

[B17] Hamilton JA Butler DM Stanton H Cytokine interactions promoting DNA synthesis in human synovial fibroblasts J Rheumatol 1994 21 797 803 8064717

[B18] Druker BJ Tamura S Buchdunger E Ohno S Segal GM Fanning S Zimmermann J Lydon NB Effects of a selective inhibitor of the Abl tyrosine kinase on the growth of Bcr-Abl positive cells Nat Med 1996 2 561 566 8616716 10.1038/nm0596-561

[B19] Buchdunger E Cioffi CL Law N Stover D Ohno-Jones S Druker BJ Lydon NB Abl protein-tyrosine kinase inhibitor STI571 inhibits in vitro signal transduction mediated by c-kit and platelet-derived growth factor receptors J Pharmacol Exp Ther 2000 295 139 145 10991971

[B20] Dewar AL Cambareri AC Zannettino AC Miller BL Doherty KV Hughes TP Lyons AB Macrophage colony-stimulating factor receptor c-fms is a novel target of imatinib Blood 2005 105 3127 3132 15637141 10.1182/blood-2004-10-3967

[B21] Carroll M Ohno-Jones S Tamura S Buchdunger E Zimmermann J Lydon NB Gilliland DG Druker BJ CGP 57148, a tyrosine kinase inhibitor, inhibits the growth of cells expressing BCR-ABL, TEL-ABL, and TEL-PDGFR fusion proteins Blood 1997 90 4947 4952 9389713

[B22] Kameda H Ishigami H Suzuki M Abe T Takeuchi T Imatinib mesylate inhibits proliferation of rheumatoid synovial fibroblast-like cells and phosphorylation of Gab adapter proteins activated by platelet-derived growth factor Clin Exp Immunol 2006 144 335 341 16634808 10.1111/j.1365-2249.2006.03067.x PMC1809657

[B23] Paniagua RT Sharpe O Ho PP Chan SM Chang A Higgins JP Tomooka BH Thomas FM Song JJ Goodman SB Lee DM Genovese MC Utz PJ Steinman L Robinson WH Selective tyrosine kinase inhibition by imatinib mesylate for the treatment of autoimmune arthritis J Clin Invest 2006 116 2633 2642 16981009 10.1172/JCI28546 PMC1564430

[B24] Sandler C Joutsiniemi S Lindstedt KA Juutilainen T Kovanen PT Eklund KK Imatinib mesylate inhibits platelet derived growth factor stimulated proliferation of rheumatoid synovial fibroblasts Biochem Biophys Res Commun 2006 347 31 35 16806061 10.1016/j.bbrc.2006.06.052

[B25] Terabe F Kitano M Kawai M Kuwahara Y Hirano T Arimitsu J Hagihara K Shima Y Narazaki M Tanaka T Kawase I Sano H Ogata A Imatinib mesylate inhibited rat adjuvant arthritis and PDGF-dependent growth of synovial fibroblast via interference with the Akt signaling pathway Mod Rheumatol 2009 19 522 529 19568828 10.1007/s10165-009-0193-x

[B26] Daniels CE Wilkes MC Edens M Kottom TJ Murphy SJ Limper AH Leof EB Imatinib mesylate inhibits the profibrogenic activity of TGF-beta and prevents bleomycin-mediated lung fibrosis J Clin Invest 2004 114 1308 1316 15520863 10.1172/JCI19603 PMC524221

[B27] Wang S Wilkes MC Leof EB Hirschberg R Imatinib mesylate blocks a non-Smad TGF-beta pathway and reduces renal fibrogenesis in vivo Faseb J 2005 19 1 11 15629889 10.1096/fj.04-2370com

[B28] Kavanaugh WM Klippel A Escobedo JA Williams LT Modification of the 85-kilodalton subunit of phosphatidylinositol-3 kinase in platelet-derived growth factor-stimulated cells Mol Cell Biol 1992 12 3415 3424 1321334 10.1128/mcb.12.8.3415-3424.1992 PMC364590

[B29] Kim G Jun JB Elkon KB Necessary role of phosphatidylinositol 3-kinase in transforming growth factor beta-mediated activation of Akt in normal and rheumatoid arthritis synovial fibroblasts Arthritis Rheum 2002 46 1504 1511 12115180 10.1002/art.10314

[B30] Mucsi I Skorecki KL Goldberg HJ Extracellular signal-regulated kinase and the small GTP-binding protein, Rac, contribute to the effects of transforming growth factor-beta1 on gene expression J Biol Chem 1996 271 16567 16572 8663331 10.1074/jbc.271.28.16567

[B31] Heldin CH Ostman A Ronnstrand L Signal transduction via platelet-derived growth factor receptors Biochim Biophys Acta 1998 1378 F79 113 9739761 10.1016/s0304-419x(98)00015-8

[B32] Xu H He Y Yang X Liang L Zhan Z Ye Y Yang X Lian F Sun L Anti-malarial agent artesunate inhibits TNF-alpha-induced production of proinflammatory cytokines via inhibition of NF-kappab and PI3 kinase/Akt signal pathway in human rheumatoid arthritis fibroblast-like synoviocytes Rheumatology (Oxford) 2007 46 920 926 17314215 10.1093/rheumatology/kem014

[B33] Hwang SY Kim JY Kim KW Park MK Moon Y Kim WU Kim HY IL-17 induces production of IL-6 and IL-8 in rheumatoid arthritis synovial fibroblasts via NF-kappab- and PI3-kinase/Akt-dependent pathways Arthritis Res Ther 2004 6 R120 128 15059275 10.1186/ar1038 PMC400429

[B34] Wang CR Shiau AL Chen SY Lin LL Tai MH Shieh GS Lin PR Yo YT Lee CH Kuo SM Liu MF Jou IM Yang CY Shen PC Lee HL Wu CL Amelioration of collagen-induced arthritis in rats by adenovirus-mediated PTEN gene transfer Arthritis Rheum 2008 58 1650 1656 18512785 10.1002/art.23517

[B35] Marone R Cmiljanovic V Giese B Wymann MP Targeting phosphoinositide 3-kinase: moving towards therapy Biochim Biophys Acta 2008 1784 159 185 17997386 10.1016/j.bbapap.2007.10.003

[B36] Hayer S Pundt N Peters MA Wunrau C Kuhnel I Neugebauer K Strietholt S Zwerina J Korb A Penninger J Joosten LA Gay S Ruckle T Schett G Pap T PI3Kgamma regulates cartilage damage in chronic inflammatory arthritis Faseb J 2009 23 4288 4298 19734303 10.1096/fj.09-135160

[B37] Yanada M Naoe T Imatinib combined chemotherapy for Philadelphia chromosome-positive acute lymphoblastic leukemia: major challenges in current practice Leuk Lymphoma 2006 47 1747 1753 17064984 10.1080/10428190600634085

[B38] Quek R George S Gastrointestinal stromal tumor: a clinical overview Hematol Oncol Clin North Am 2009 23 69 78 viii 19248971 10.1016/j.hoc.2008.11.006

[B39] Eklund KK Joensuu H Treatment of rheumatoid arthritis with imatinib mesylate: clinical improvement in three refractory cases Ann Med 2003 35 362 367 12952023 10.1080/07853890310001339

[B40] Miyachi K Ihara A Hankins RW Murai R Maehiro S Miyashita H Efficacy of imatinib mesylate (STI571) treatment for a patient with rheumatoid arthritis developing chronic myelogenous leukemia Clin Rheumatol 2003 22 329 332 14576993 10.1007/s10067-003-0716-3

[B41] Koyama K Hatsushika K Ando T Sakuma M Wako M Kato R Haro H Sugiyama H Hamada Y Ogawa H Nakao A Imatinib mesylate both prevents and treats the arthritis induced by type II collagen antibody in mice Mod Rheumatol 2007 17 306 310 17694264 10.1007/s10165-007-0592-9

[B42] Ando W Hashimoto J Nampei A Tsuboi H Tateishi K Ono T Nakamura N Ochi T Yoshikawa H Imatinib mesylate inhibits osteoclastogenesis and joint destruction in rats with collagen-induced arthritis (CIA) J Bone Miner Metab 2006 24 274 282 16816921 10.1007/s00774-006-0684-1

[B43] Leder C Ortler S Seggewiss R Einsele H Wiendl H Modulation of T-effector function by imatinib at the level of cytokine secretion Exp Hematol 2007 35 1266 1271 17560008 10.1016/j.exphem.2007.04.016

[B44] Taylor JR Brownlow N Domin J Dibb NJ FMS receptor for M-CSF (CSF-1) is sensitive to the kinase inhibitor imatinib and mutation of Asp-802 to Val confers resistance Oncogene 2006 25 147 151 16170366 10.1038/sj.onc.1209007

[B45] Juurikivi A Sandler C Lindstedt KA Kovanen PT Juutilainen T Leskinen MJ Maki T Eklund KK Inhibition of c-kit tyrosine kinase by imatinib mesylate induces apoptosis in mast cells in rheumatoid synovia: a potential approach to the treatment of arthritis Ann Rheum Dis 2005 64 1126 1131 16014680 10.1136/ard.2004.029835 PMC1755598

